# A Microfluidic Eye Facsimile System to Examine the Migration of Stem-like Cells

**DOI:** 10.3390/mi13030406

**Published:** 2022-03-02

**Authors:** Stephen Ryan Mut, Shawn Mishra, Maribel Vazquez

**Affiliations:** 1 Department of Biomedical Engineering, Rutgers, The State University of New Jersey, 599 Taylor Rd, Piscataway, NJ 08854, USA; sm2116@scarletmail.rutgers.edu; 2 Regeneron, 777 Old Saw Mill River Rd, Tarrytown, NY 10591, USA; shawnmishra2007@u.northwestern.edu

**Keywords:** electric fields, chemotaxis, retina, transplantation

## Abstract

Millions of adults are affected by progressive vision loss worldwide. The rising incidence of retinal diseases can be attributed to damage or degeneration of neurons that convert light into electrical signals for vision. Contemporary cell replacement therapies have transplanted stem and progenitor-like cells (SCs) into adult retinal tissue to replace damaged neurons and restore the visual neural network. However, the inability of SCs to migrate to targeted areas remains a fundamental challenge. Current bioengineering projects aim to integrate microfluidic technologies with organotypic cultures to examine SC behaviors within biomimetic environments. The application of neural phantoms, or eye facsimiles, in such systems will greatly aid the study of SC migratory behaviors in 3D. This project developed a bioengineering system, called the μ-Eye, to stimulate and examine the migration of retinal SCs within eye facsimiles using external chemical and electrical stimuli. Results illustrate that the imposed fields stimulated large, directional SC migration into eye facsimiles, and that electro-chemotactic stimuli produced significantly larger increases in cell migration than the individual stimuli combined. These findings highlight the significance of microfluidic systems in the development of approaches that apply external fields for neural repair and promote migration-targeted strategies for retinal cell replacement therapy.

## 1. Introduction

**Figure 1:**
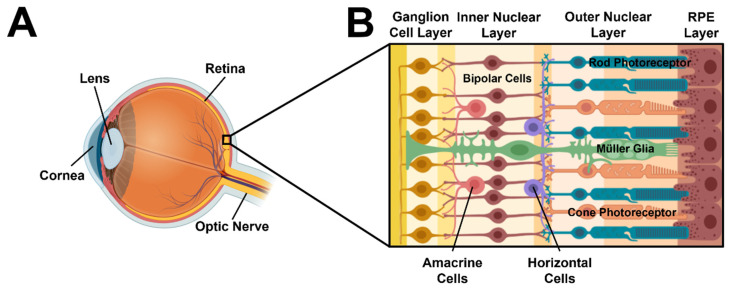
Schematic representation of the human retina at the eye posterior. (**A**) Depiction of the human eye showing the cornea, lens, and retina. (**B**) A schematic cross-section of healthy retina that consists of (from right to left): a layer of non-neural, retinal pigmented epithelium (RPE), the outer nuclear layer of primary retinal cells (rod and cone photoreceptors), an inner nuclear layer of secondary or interneurons (amacrine, horizontal, and bipolar cells), and the ganglion cell layer (ganglion cells). Axons of the retinal ganglia form the optic nerve that then delivers signals to the brain for vision.

An unprecedented 500 million adults, worldwide, will be visually impaired by the year 2050 [1]. Vision loss in mature and aging adults is prevalent in developed countries [2,3,4] and often arises from dysfunction in the retina, a photosensitive tissue that lines the posterior of the eye and connects to the brain. The human retina contains over 100 million neural cells that synapse to enable vision (reviewed in [5,6]), as shown in Figure 1. Photoreceptor neurons of the outer nuclear layer (ONL) absorb and transduce light into electrical signals via the processes of phototransduction. These signals are then transmitted across different neuronal groups in the inner nuclear layer (INL) and ganglion cell layer (GCL) to produce images of objects in the brain. However, since retinal neurons cannot self-repair, cell damage rapidly propagates synaptic dysfunction [7,8] to result in progressive vision loss from complex disorders, such as age-related macular degeneration [9,10], diabetic retinopathy [11], and retinitis pigmentosa [12].

Regenerative medicine offers the exciting promise to restore vision through cell replacement therapy, where stem and progenitor-like cells (SCs) are transplanted into the retinal host to replace damaged and/or degenerated neurons (reviewed in [13,14,15,16]). A classical model of functional integration relies upon transplanted cells to perform several complex behaviors [17,18,19] including: (i) surviving surgical insertion into a damaged, adult host [20]; (ii) Infiltrating endogenous neural networks to achieve desired cellular positioning [21,22]; (iii) Differentiating appropriately into targeted neuronal cell type(s) [23]; and (iv) Initiating new synapses with functional, native cells to re-establish vision [24,25,26].

The poor infiltration of replacement cells within the adult host remains a principal challenge [27,28,29,30], as numerous projects have reported very small numbers of viable cells [31] and insufficient cell migration into targeted retinal laminae [21,27]. Biomedical groups have sought to overcome these limitations by deriving either replacement SCs with increasingly specialized lineage(s) [32,33,34] or cells that transiently express proteins to aid motility [35,36,37]. In tandem, bioengineering projects have developed in vitro platforms and ex vivo platforms to study SC migration towards different signaling fields [38,39,40,41,42]. However, manipulation of motility in transplanted cells requires an integration of both approaches, as controlled studies of specialized SC responses to cues from 3D, physiochemical conditions of the adult eye remain underexplored.

This project developed the μ-Eye, a microfluidic eye facsimile system that uses external chemical and electrical stimuli to guide the migration of SCs within spherical biomimetic environments. The system is comprised of microfluidic reservoirs of chemoattractants and media, microfabricated electrodes, and hydrogel-based eye facsimiles. Experiments applied external electrical fields and chemical gradients to illustrate >95% cell survival overnight, as well as dramatic differences in the numbers of motile SCs, their penetration depths, and distributions within eye facsimiles. Moreover, results illustrate that SCs migrated significantly larger distances, and in larger numbers, when exposed to combined electrical and chemotactic fields than when compared to individual stimulus alone. Such exciting findings highlight the abilities of the μ-Eye system to investigate the therapeutic use of external fields in cell replacement therapy and to explore new, migration-targeted approaches to the transplantation of highly specialized SC populations.

## 2. Materials and Methods

### 2.1. Cell Culture

Replacement stem and progenitor-like cells (SCs) were modeled using R28 cells (Cat. No. EUR201, Kerafest, Inc., Boston, MA, USA), derived from a post-natal day six, rat retinal culture widely used for in vivo and in vitro testing (reviewed in [43]). Cells were incubated in conventional mammalian culture conditions of 37 °C, 5% CO_2_, and 95% humidity and maintained in complete media comprised of Dulbecco’s Modified Eagle’s Medium (DMEM; Cat. No. 30-2002, ATCC, Manassas, VA, USA) supplemented with 10% fetal bovine serum (FBS; Cat. No. 26140, Thermo Fisher Scientific, Waltham, MA, USA), 100 mg/mL penicillin-streptomycin (Cat. No. 15070063, Thermo Fisher Scientific, Waltham, MA, USA) and 4.5 mg/mL L-glutamine (Cat. No. 25030024, Thermo Fisher Scientific, Waltham, MA, USA). Cells were passaged regularly to ensure 85–95% confluency within tissue flasks. Briefly, cells were washed twice with Dulbecco’s phosphate buffered saline (1× DPBS; Cat. No. 21-031-CV, VWR, Radnor, PA, USA) and then detached from flask surfaces using Accutase solution (Cat. No. AT104-500, ICT Inc., Glendale, CA, USA). Cells were then centrifuged at 125× *g* for three minutes, re-suspended in media, and re-plated onto tissue flasks at approximate cell densities of 1 × 10^6^ to 5 × 10^6^ cells/mL.

### 2.2. Fluorescent Labeling and Immunocytochemical (ICC) Staining 

Cells were fluorescently labeled with CellTracker^TM^ (Cat. No. C2925, Thermo Fisher Scientific, Waltham, MA, USA) to aid in cell visualization. A 10.8-μL stock solution of Cell Tracker was suspended in dimethyl sulfoxide (DMSO; Cat. No. 196055, MP Biomedicals LLC, Solon, OH, USA) and then reconstituted (1:1000 dilution) to a 10-mL working volume using serum free medium (DMEM without FBS) as the diluting agent. Tissue flasks were incubated with Cell Tracker solution for 45 min at physiological conditions (37 °C, 5% CO_2_, and 95% humidity). Following incubation, the Cell Tracker solution was aspirated from the tissue flasks and cells were rinsed twice with 1× DPBS solution prior to experimentation.

**Figure 2:**
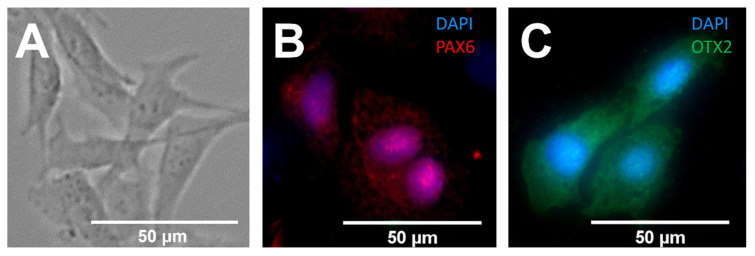
Expression of markers for retinal encoding genes within a cultured cell model of retinal precursors. (**A**) Bright field image of cells upon a control, cell culture surface. (**B**,**C**) Immunocytochemical (ICC) labeling of genetic markers within model cells: (**B**) Paired Homeobox gene 6 (PAX6: Red), an early retinal progenitor marker observed in the cell nuclei and cyto-sol and (**C**) Orthodenticle Homeobox 2 (OTX2: Green), a photoreceptor precursor marker ex-pressed in both the nucleus and cytosol. Cell nuclei are stained with DAPI (Blue). Scale = 50 μm.

Cell expression of markers for Paired homeobox 6 (PAX6) (Cat. No. 42-6600, Thermo Fisher, Waltham, MA, USA) and Orthodenticle Homeobox 2 (OTX2) (Cat. No. 13497-1-AP, Thermo Fisher, Waltham, MA, USA) were examined via immunocytochemistry (ICC) (Figure 2), as consistent with previous studies from our group [40,44]. Briefly, cells were seeded into borosilicate well plates (Cat. No. 155411, Thermo Fisher, Waltham, MA, USA) on Day 1 at a variable density (3 × 10^4^ to 3 × 10^5^ cells/well). For each experiment, cells were plated from the same culture flask to indicate the same differentiation status and were stained with PAX6 and OTX2 in parallel wells at the same timepoints. On Day 2, cells were fixed in 4% paraformaldehyde (PFA) for 20 min and then washed three times with 1× DPBS for 5 min each. A blocking buffer consisting of normal donkey serum (Cat. No. 017-000-121, Jackson ImmunoResearch Laboratories, West Grove, PA, USA), Triton X (Cat. No. 200002-540, VWR, Radnor, PA, USA), and bovine serum albumin (BSA; Cat. No. 97061-420, VWR, Radnor, PA, USA) was added to each well for 1 h to permeabilize cells for staining. Following subsequent DPBS washes, primary antibodies for PAX6 and OTX2 were diluted (1:100) in Dako antibody dilutant solution (Cat. No. S3022, Agilent, Santa Clara, CA, USA) and added to parallel wells of cells. Negative control wells did not add the primary antibody to treated cells and did not demonstrate non-specific binding (data not shown). After overnight incubation at 4 °C on Day 3, primary antibodies were removed and washed with 1× DPBS three times at 5 min each. Secondary antibodies (Cat. No. 711-025-152, Jackson ImmunoResearch Laboratories, West Grove, PA, USA) were diluted in Dako (1:200) and added to each well for 2 h at room temperature (RT). DAPI nuclear stain (Cat. No. D1306, Thermo Fisher, Waltham, MA, USA) was diluted in 1× DPBS (38 nM) and was added to each well for 5 min and then washed twice with 1× DPBS and once with ultrapure water (5 min per wash).

### 2.3. Preparation of Cells

Cells were suspended within an artificial basement membrane (Matrigel; 8–11 mg/mL; Cat. No. 356230, Corning Inc., Corning, NY, USA) for testing. Stock vials of Matrigel were thawed overnight on ice at 4 °C while all pipette tips, glassware, and associated tools were maintained on ice to prevent premature gelation. The Matrigel solution was diluted with complete medium to a working concentration of 5 mg/mL. Tests then mixed a 90-µL aliquot of 5 mg/mL Matrigel with a 10-µL volume of cells that were re-suspended in complete media at a cell density of approximately 5 × 10^6^ cells/mL.

### 2.4. External Stimuli

Electric fields (EF) of 100 mV/mm direct current were applied using a NI myDAQ data acquisition device (National Instruments, Austin, TX, USA) and the NI Arbitrary Waveform Generator (National Instruments, Austin, TX, USA). EF stimulation was first verified by digital multimeter (Model 77-IV, Fluke Corporation, Everett, WA, USA) and then applied across the eye facsimile for a total of 5 min. Chemical fields were applied using solutions of 100-ng/mL of Stromal Cell Derived Factor (SDF-1α; Cat. No. SRP4388, Sigma-Aldrich, St. Louis, MO, USA) for 12 h or overnight testing, as done previously by our group [39,45].

### 2.5. Eye Facsimiles

Facsimiles were synthesized using hydrogel beads of alginate, a natural polysaccharide commonly used in biomaterial applications of drug release and ophthalmology [46,47,48]. Facsimiles were produced with an approximate diameter of 3 mm to model the adult murine eye and were synthesized by mixing solutions of sodium alginate (Cat. No. W201502, Sigma-Aldrich, St. Louis, MO, USA) with two individual crosslinking agents: calcium lactate (Cat. No. L4388, Sigma-Aldrich, St. Louis, MO, USA) and zinc chloride (Cat. No. 208086, Sigma-Aldrich, St. Louis, MO, USA). Briefly, 2% *w*/*v* sodium alginate powder was dissolved in deionized water and mixed at 400 rpm on a heated stir plate at 95°C. After 1 h of mixing, the alginate solution was removed from the hotplate and desiccated for 15 min to remove air bubbles. Then, using a 1-mL Luer-slip syringe with needle (Cat. No. 4010-200V0; Cat. No. 305564, VWR, Monroeville, PA, USA), the 2% alginate solution was added, dropwise, to a 5% gelling solution of calcium lactate that was dissolved in deionized water. Facsimiles were formed in the gelling solution and centrifuged at 400 rpm for 1 h to facilitate complete gelation. This step was independently repeated for crosslinking sodium alginate with zinc chloride. Following centrifugation, eye facsimiles were stored in serum free DMEM and maintained at 4 °C prior to experimentation.

### 2.6. Molecular Transport across Eye Facsimiles

**Figure 3:**
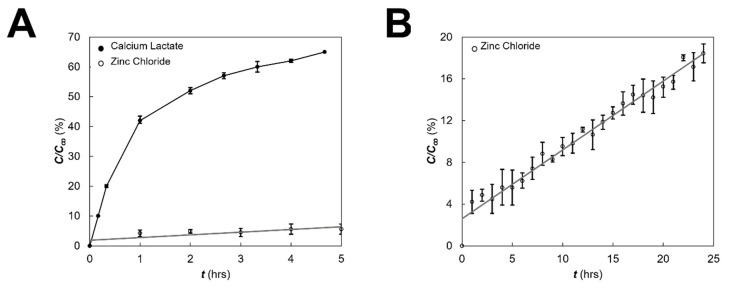
Measurement of diffusion outwards from eye facsimiles. (**A**) Time-dependent release of fluorescent marker (dextran; 10 kDa) within alginate crosslinked with calcium lactate and with zinc chloride as measured by the ratio of the instantaneous concentration (*C*) to the equilibrium concentration (*C*_∞_) in solution. (**B**) Chemical release from alginate-zinc chloride facsimiles over longer, 25-h time periods. All data were acquired in triplicate (*n* = 3) per condition and reported as mean values with standard deviation.

Transport across eye facsimiles was estimated using experiments to measure outward diffusion of dextran molecules (10 kDa MW, Cat. No. FD20S, Sigma-Aldrich, St. Louis, MO, USA) from hydrogel beads, a priori, as described previously by our group [49]. This fluorescent molecule was chosen for its similar molecular weight and diffusivity to SDF-1α in aqueous solution [50,51]. Briefly, synthesized facsimiles were immersed in a dextran solution for 24 h and then preserved with 10% (*w*/*v*) sodium citrate solution. The average fluorescent intensity of sample aliquots (*n* = 5) of this solution was measured via fluorimeter (Cytation 5 Image Reader, BioTek Instruments Inc., Winooski, VT, USA) to estimate the dextran loading concentration, *C*_∞_, within hydrogel beads. Dextran-loaded eye facsimiles were then transferred to 1× DPBS solutions to measure outward diffusion of dextran to the surrounding solution over time. The average fluorescent intensity of aliquots surrounding the dextran-filled facsimiles (*n* = 5) were measured via fluorimeter and normalized with respect to the initial loading concentration (*C*). Figure 3 illustrates the data used to model the chemical release profile of eye facsimiles.

As shown, eye facsimiles crosslinked with calcium lactate exhibited a molecular release of dextran that was initially linear, but rapidly increased to approach 65% of the loading concentration, *C*, after a few hours. This rapid, sigmoidal release was consistent with previous studies using calcium-based crosslinking of alginates (reviewed in [52]) but was undesirable for the multi-day testing desired for the μ-Eye system. By contrast, eye facsimiles crosslinked with zinc chloride produced a much slower, linear release of dextran over 24 h that was also consistent with the reported literature [53,54]. This facsimile therefore provided a chemical environment that facilitated the measurement of small changes in chemical concentration for μ-Eye testing.

### 2.7. Finite Element Modeling

A finite element model (FEM; COMSOL Multiphysics 5.3a, COMSOL Inc., Burlington, MA, USA) was used to describe the distributions of electric potential and chemical concentration across facsimiles of the μ-Eye. The electrostatics module with a steady current density, (*i*), defined by Equation (1), was used to determine the charge accumulation within the system, (*Q*). The volume integral of the charge density, (*ρ*), given by Equation (2) was then related to the electric field, (*E*), via alginate permittivity, *ε* [55], as per Equation (3).

(1) $$0=\vec{\nabla }\cdot i$$

(2) $$Q=\int_{V}^{}\rho dV$$

(3) $$Q=\varepsilon \oint_{s}^{}\vec{E}\cdot \vec{n}\;dA$$

The total charge accumulation, (*Q*), was related to the electric field, (*E*), via the Divergence theorem, as shown in Equations (4) and (5). The electric potential, (*ϕ*), was then correlated to the electric field (*E*) in Equation (6) and expressed in terms of the charge density, (*ρ*), in Equation (7).

(4) $$\int_{V}^{}\rho dV=\varepsilon \int_{V}^{}\nabla \cdot \vec{E}\;dV$$

(5) $$\nabla \cdot \vec{E}=\frac{\rho }{\varepsilon }$$

(6) $$∆\phi =-\int \vec{E}dl$$

(7) $$\frac{\rho }{\varepsilon }=-\nabla^{2}\phi$$

Lastly, Equation (7) was solved numerically to determine the distribution of the electrical potential, (*ϕ*), across eye facsimiles.

Chemical stimulation across eye facsimiles was modeled using The Transport of Diluted Chemical Species module. This model is governed by Fick’s Second Law of diffusion, which relates time, (*t*), and spatial distribution, (*r*), of concentration, (*C*), via the diffusivity constant, (*D*), as shown in Equation (8).

(8) $$\frac{\partial C}{\partial t}=D\frac{1}{r^{2}}\frac{\partial }{\partial r}\left(r^{2}\frac{\partial C}{\partial r}\right)$$

A numerical solution to Equation (8) was conducted over the experimental time range of *t* = 0–12 h For these solutions, a value of 1.6 × 10^−6^ cm^2^/s was used for SDF-1α diffusivity, as previously measured by our group [56]. An initial growth factor concentration at *t* = 0 h, (*C*_o_), was specified at the chemical reservoir (source) that maintained a 100-ng/mL solution of SDF-1α. Lastly, an initial concentration of 0-ng/mL was specified for the opposite media reservoir (sink).

### 2.8. Fabrication of System Components

**Figure 4:**
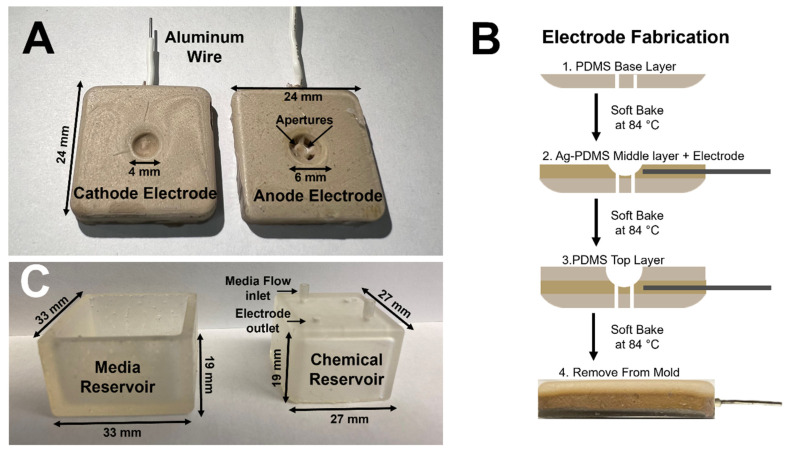
Fabrication of μ-Eye components. (**A**) Top view of the cathode and anode chambers consisting of aluminum wires and spherical depressions within each. Media apertures labeled. Dimensions and system component features labeled. (**B**) Schematic representation of the soft lithography process used to fabricate electrodes. (**C**) Image of 3D printed chemical and media reservoirs. Electrode outlets and media flow inlets labeled.

The μ-Eye system was fabricated using 3D printed and microfabricated components, which include a media reservoir, chemical reservoir, cathode chamber, and anode chamber, as shown in Figure 4. The dimensions of each piece were designed to accommodate an eye facsimile of an enucleated, adult mouse eye.

The anode and cathode electrodes (Figure 4A) were fabricated via conventional soft lithography (described previously by our group and others [57], reviewed in [58]) with external dimensions of 24 × 24 mm^2^ and a thickness of 4 mm each. The inner surface of the anode contains a 6-mm spherical depression and four equally spaced apertures to maintain fluidic contact with media in the chemical and media reservoirs. The cathode inner surface contains a smaller, 4-mm spherical depression. Electrodes were manufactured in three separate layers using conventional soft lithography, as described previously by our group [49] and shown in Figure 4B. 3D printed imprint molds for each electrode were developed using the dimensions described above. The first layer of each electrode was formed from a polymer solution of polydimethylsiloxane (PDMS; Cat. No. 1020992-312, VWR, Radnor, PA, USA) synthesized by mixing a 9:1 volume ratio of the commercial elastomer and curing agent. The mixture was homogenized and desiccated for 15 min to remove air bubbles. Approximately 1-mL of PDMS was poured into the imprint mold and oven-cured (Cat. No. ED056UL-120V, Binder GmbH, Tuttlingen, Germany) at 84 °C for 10 min. Afterwards, the second layer of each electrode was produced using a silvered-PDMS (Ag-PDMS) solution developed by mixing PDMS with 80% *w*/*v* silver nanospheres (Cat. No. 327085, Sigma-Aldrich, St. Louis, MO, USA), as done previously by our group [39]. This mixture was similarly homogenized and desiccated for 15 min and then poured atop the first layer of PDMS. Insulated aluminum wires were embedded within the Ag-PDMS layer and the entire mold was then re-inserted into the oven for curing. Finally, a third layer of PDMS was added atop the second layer of Ag-PDMS and re-cured for 15 min. A precision knife with fine blade was then used to carefully remove the tri-layered electrode chambers from the imprint molds.

The media and chemical reservoir chambers (shown in Figure 4C) were 3D printed (Formlabs Form 2 Stereolithography printer (Cat. No. FH-CU-01, Dynamism Inc., Chicago, IL, USA) using a colorless v4 resin (Cat. No. RS-F2-GPCL-04, Dynamism Inc., Chicago, IL, USA) at a layer printing resolution of 25 µm. The 3D printed reservoirs were then cured for 10 min using a Formlabs Form Cure^TM^ UV curing system (Cat. No. FH-CU-01, Dynamism Inc., Chicago, IL, USA) with a UV lamp (λ_M_ = 405 nm). The chemical reservoir has a 14-mL volume capacity and contains a square base with dimensions of 33 × 33 mm^2^, while the media reservoir has a 22-mL volume capacity and contains a smaller square base with dimensions of 27 × 27 mm^2^. Both reservoirs were produced with a height of 19 mm. In addition, the chemical reservoir was manufactured with four apertures of 2-mm diameter that were drilled into the top side to provide outlets for electrode wires, as well as media ports for injecting media and chemical solutions.

Prior to experimentation, electrodes and reservoirs were bathed in a 70% ethanol solution and mixed at 400 RPM on a stir plate for 30 min. Electrodes and reservoirs were then transferred to a biosafety cabinet (Cat. No. 1300 Series A2, Thermo Fisher Scientific, Waltham, MA, USA) and rinsed with autoclaved ultrapure water, dried by non-abrasive tissues (Cat. No. 34155, VWR, Radnor, PA, USA), and sterilized under UV light for 30 min.

### 2.9. Cryosection and Imaging

Eye facsimiles treated with external fields were fixed in 4% paraformaldehyde (PFA) solution supplemented with 50 mM sucrose and 10 mM calcium chloride following stimulation. Eye facsimiles (*n* = 7–11, per condition) were then transferred from the fixation solution, placed into 2 mL of optimal cutting temperature compound solution (OCT solution; Cat. No. 23730571, Fisher Scientific, Waltham, MA, USA), and left overnight at 4 °C. Afterwards, eye facsimiles were transferred to cryomolds filled with OCT and frozen in a −80 °C freezer. The frozen samples were removed from the cryomold and sectioned into 20-μm-thick slices using a cryostat (Cat. No CM1950, Leica Biosystems, Nußloch, Germany). The sectioned samples were then mounted onto coated glass slides (ProLong Gold Antifade Mountant; Cat. No. P36930, Thermo Fisher Scientific, Waltham, MA, USA) and optically imaged to measure penetration depth, or infiltration, of fluorescently labeled cells within eye facsimiles.

A multiphase, inverted microscope (DMi8, Leica Instruments, Nußloch, Germany) was used to measure the migration of cells within different radial depths of eye facsimiles using a 20X objective (Cat. No. 11506243, Leica Instruments, Nußloch, Germany) and built-in CCD camera (DFC7000 GT, Leica Instruments, Nußloch, Germany).

### 2.10. Data Analyses and Statistics

Data to describe cell migration within eye facsimiles was analyzed after chemotactic stimulation (SDF), electric field stimulation (EF), and electro-chemotactic stimulation (EC). Images of fluorescence intensity within sectioned facsimile samples of each treatment group (*n* = 7–11 per stimulus condition) were converted into 8-bit data (1:255) using ImageJ software (National Institutes of Health, Bethesda, MD, USA). Average, total numbers of motile cells within facsimiles, N_T_, and cell distances traveled within eye facsimiles were analyzed using the ImageJ Analyze Particles Package. Final cell positions were denoted as penetration depths, PD, and defined in Equation (9).

(9) $$\mathrm{PD}=\left|R_{\mathrm{Surf}}-R_{\mathrm{Final}}\right|$$

where *R*_Surf_ is the radius at the surface interface of the eye facsimile and initial population of SCs, and *R*_Final_ is the radial position of motile cells at the end of the experimental duration. The range of cell movement within eye facsimiles was defined as *R* to denote the difference between maximum and minimum PD values for each experimental condition, as per Equation (10).

(10) $$R=\left|{\mathrm{PD}}_{Max}-{\mathrm{PD}}_{Min}\right|$$

Note cells that failed to penetrate eye facsimiles after stimulation were excluded from analyses.

The statistical significance was measured across all conditions using GraphPad (v9; Graphpad Software Inc., San Diego, CA, USA). The D’Agostino-Pearson Omnibus test was first conducted (confidence level, α = 0.05) to assess the normal distribution of data collected form all stimulus conditions [59]. These tests identify deviations from parametric data when the parameter, *p*_NORM_, is larger than 0.05. Second, the Kruskal-Wallis ranked sum variance and Dunn post hoc test (at confidence level, α = 0.05) were applied to determine statistical significance across groups, as described previously [60]. Significance was denoted by a single asterisk, (*), for *p* < 0.05, a double asterisk (**), for *p* < 0.01, and a triple asterisk (***) for *p* < 0.001.

## 3. Results

### 3.1. μ-Eye Design and Operation

**Figure 5:**
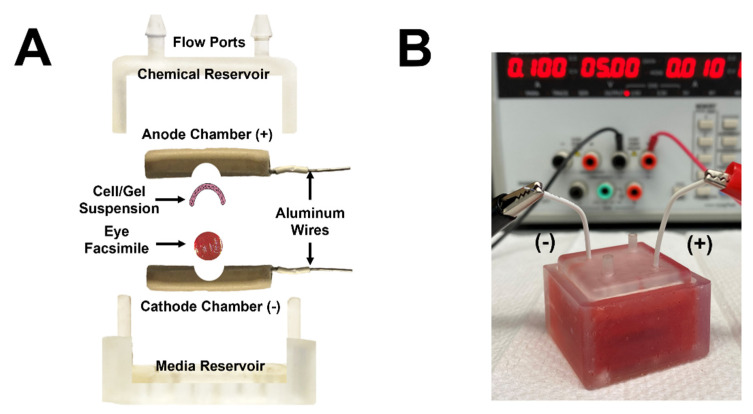
Overview of the μ-Eye system used to apply external chemical and electrical fields across eye facsimiles. (**A**) Exploded view of the μ-Eye system components: chemical reservoir (with flow ports), media reservoir, anode, and cathode electrodes. Enclosed within the electrode assembly is an eye facsimile and gel/suspension of cells. (**B**) Representative image of a fully assembled μ-Eye system during testing.

The μ-Eye system is comprised of two fluidic reservoirs that contain a chemoattractant and media solutions and one electrode assembly comprised of a cathode and anode chamber, as shown in Figure 5. The dimensions of each component were designed to accommodate an eye facsimile synthesized to the size of an enucleated, adult murine eye. The top part of the μ-Eye consists of a chemical reservoir with two flow ports for loading chemoattractant solutions, while the bottom contains a fluidic reservoir of cell media. The electrode assembly lies within the reservoirs and houses the eye facsimile. The inner surface of each electrode chamber is fabricated with a circular depression to hold one eye facsimile and a suspension/gel containing SCs for testing. When closed, the chambers adjoin the eye facsimile and SC-laden mixture to facilitate cell migration into facsimiles.

The operation of the μ-Eye is performed in four steps. First, the bottom reservoir is filled with the media solution. Second, the eye facsimile and SC suspension/gel are positioned within the electrode chambers and closed. Third, the coupled electrode assembly is placed into the media reservoir. Fourth, the chemical reservoir is placed over top of the system and filled with chemoattractant solution until the liquid level reaches its maximum. Apertures within the chambers and reservoirs create fluidic contact with the eye facsimile such that the conjoined components create a closed environment wherein electrical and chemical stimulation are applied. This study used the μ-Eye to apply three types of external fields to stimulate SC migration into facsimiles: (i) chemotactic concentration gradients (SDF); (ii) electrical fields (EF) of direct current; and (iii) combinatory electro-chemotactic (EC) stimuli that superimposed SDF and EF fields during testing.

### 3.2. Modeling and Validation of External Fields

**Figure 6:**
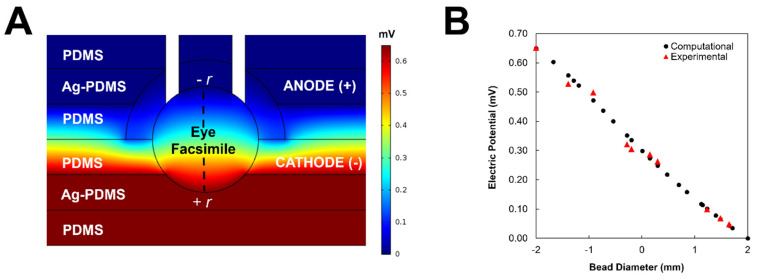
Model and validation of electric potentials across the μ-Eye system. (**A**) Finite element model of electric potentials within the electrode assembly. Low values are denoted by dark blue and high values appear in dark red. Dashed line represents facsimile diameter. (**B**) Numerical representation of the electric potential across the electrode assembly (black circles) alongside experimental values (red triangles) recorded by multimeter.

**Figure 7:**
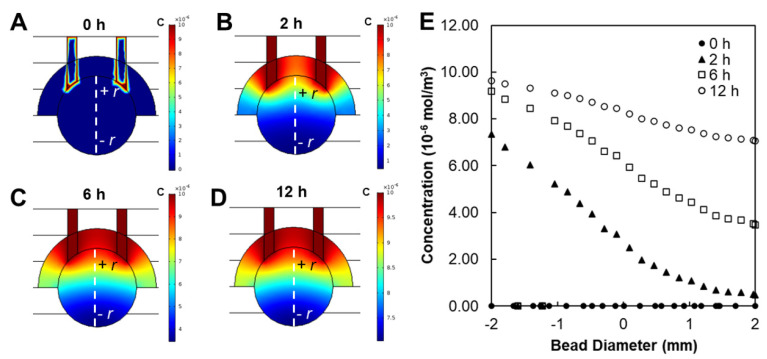
Modeling of chemical transport across the μ-Eye system. Finite element model of concentration gradients within eye facsimiles (dashed line) at (**A**) 0 h, (**B**) 2 h, (**C**) 6 h, and (**D**) 12 h. (**E**) Representation of concentration profiles across the μ-Eye at selected time points from 0–12 h.

Prior to testing within the μ-Eye, electrical and chemical fields applied across eye facsimiles were modelled computationally and verified experimentally. Figure 6 illustrates that a uniform EF was developed across the facsimile diameter (dashed line), where areas of high (bright red) and low (dark blue) electrical potential are modeled throughout the system. Computation of electrical potentials in the μ-Eye estimated a linear decrease from anode to cathode across the facsimile, which was validated using multi-meter readings across multiple points of the closed electrode assembly (as per Figure 6B). Similarly, Figure 7 depicts the modeling of chemical fields across the system, where areas of high concentration are shown in bright red and regions of low (or zero) concentration are denoted in dark blue. Transport within the facsimile was modelled by using the temporal evolution of concentration gradients. Values of chemical concentration across facsimiles were then experimentally validated over time by measuring the intensity of a well-studied fluorescent molecule synthesized into hydrogels a priori (as per Figure 3).

### 3.3. Migration of SCs within Eye Facsimiles 

**Figure 8:**
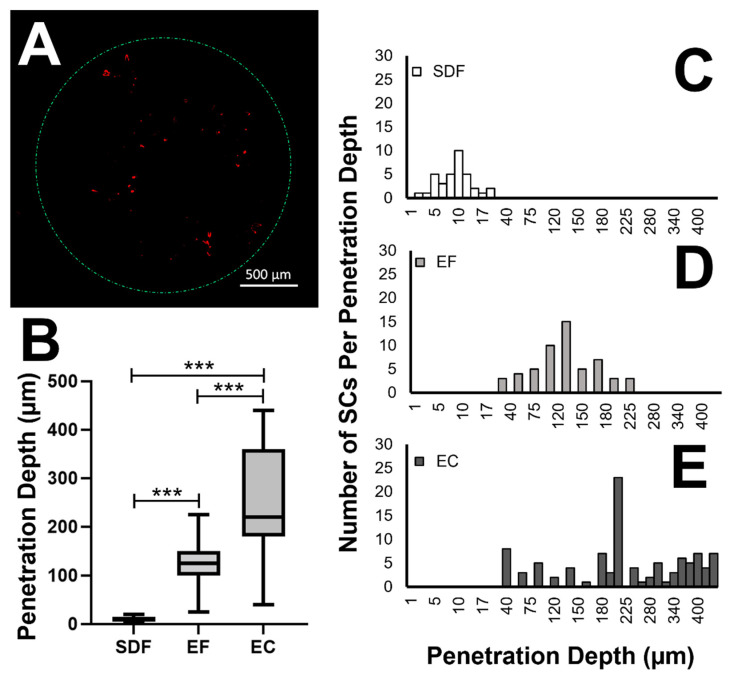
Migration of cells into eye facsimiles in response to externally applied chemical fields (SDF), electric fields (EF), and electro-chemotactic (EC) fields. (**A**) Representative image of cells (red) within eye facsimiles upon stimulation. (**B**) Average penetration depths (PDs) of motile cells within eye facsimiles in response to SDF, EF, and EC fields. Box plots represent the average (horizontal bar), quartile distribution (box), and range (vertical bars) of all data sets per condition. Numbers of motile cells per penetration depth within eye facsimiles in response to (**C**) SDF, (**D**) EF, and (**D**) EC stimulation. Experiments were performed in triplicate for each stimulus (*n* = 7–11 per condition), and statistical significance across averages is denoted by (***) for *p* < 0.001.

The migration of SCs within facsimiles of the μ-Eye system was assessed using three metrics: (i) total, average number of motile cells within facsimiles post-stimulus, N_T_; (ii) average penetration depth, or radial distance traveled within eye facsimiles, PD; and (iii) distribution of motile SCs within facsimiles defined by normality values, *p*_NORM_, total migration range, R_T_, and migration quartile, Q. Figure 8 illustrates the different numbers of motile cells per penetration depth in response to chemotactic fields (SDF), electric fields (EF), and combined electro-chemotactic (EC) fields. The values of the parameters studied are summarized in Table 1.

As shown, the average, total number of SCs that migrated into eye facsimiles when stimulated by SDF concentration gradients was ^SDF^N_T_ = 35 ± 2 cells. These cells migrated total distances between 2 μm and 20 μm from the outer facsimile surface, for a total range of ^SDF^R_T_ = 18 μm, and exhibited an average penetration distance of ^SDF^PD_AVG_ = 9.8 μm ± 4.0 μm. Similarly, the average, total number of SCs that migrated into eye facsimiles as a result of EF stimulus was ^EF^N_T_ = 55 ± 4 cells. These SCs migrated total distances between 25 μm and 225 μm for a range of ^EF^R_T_ = 200 μm and exhibited an average penetration distance of ^EF^PD_AVG_ = 123 μm ± 50 μm. By contrast, the average, total number of motile SCs due to EC stimulation was ^EC^N_T_ = 101 ± 4 cells, which migrated between 40 μm and 440 μm for a range of ^EC^R_T_ = 400 μm. SCs that became motile in response to EC fields exhibited an average penetration distance of ^EC^PD_AVG_ = 248 μm ± 121 μm within eye facsimiles. Statistical significance was measured across all conditions (*p* < 0.001). In addition, the distributions of motile cells were examined for normality or Gaussian properties. As seen in Table 1, the final positioning of SCs within eye facsimiles exhibited PD values that were Gaussian for both SDF and EF stimulation, with normality values of ^SDF^*p*_NORM_ = 0.12 and ^EF^*p*_NORM_ = 0.88, respectively. By contrast, the distribution of SC within facsimiles post-EC stimulation was non-Gaussian, with a value of ^EC^*p*_NORM_ < 0.001.

Cell positioning, or distribution within eye facsimiles, was further analyzed to highlight sub-groups of SCs with extreme motility in response to stimulus conditions. For the analysis shown in Table 2, cells that responded with low motility were defined as SCs with values of PD in the bottom quartile, Q_1_, or within the lowest 25% of the measured range, R. Conversely, cells denoted as having elevated motility produced PD values within the top quartile, Q_4_, or highest 25% of the measured range. Of the average total number of motile SCs produced by chemotactic fields (^SDF^N_T_ = 35 ± 2), 20% exhibited low motility (Q_1_) and 8.6% illustrated elevated motility (Q_4_). When using EF stimulation (^EF^N_T_ = 55 ± 4 cells), 21.8% of cells exhibited low motility (Q_1_) and 10.9% exhibited elevated motility (Q_4_). Lastly, EC fields (^EC^N_T_ = 101 ± 4 cells) stimulated 21.8% of cells to exhibit low motility (Q_1_) and 28.7% of cells to exhibit elevated motility (Q_4_). In all experiments, the portion of SCs with average motility (Q_2_, Q_3_) decreased from SDF, to EF, to EC, while sub-groups with the lowest motility (Q_1_) remained approximately unchanged.

## 4. Discussion

Microfluidic technologies are well-suited for the study of retinal degenerative diseases, as well as the development of novel therapies, by developing models that mimic the physiological environment at the retinal microscale. Organ-on-a-chip platforms (reviewed in [61,62]) have been recently developed by multiple groups using retinal stem and progenitor cells (SCs) [63,64,65,66] or organoids [67,68,69,70] to recapitulate the retinal cellular niche in both healthy and disease states. Some existing systems use 3D, organotypic systems that integrate explanted retina to measure tissue viability and cytotoxicity [71], while others examine the delivery of therapeutic compounds [41,42]. However, few 3D platforms have been applied to the study of cell replacement therapies, or transplantation, which offer exciting possibilities to treat adult retinopathies [72]. While transplantation relies upon SC abilities to differentiate and infiltrate host tissue (reviewed in [73]), few projects have examined the motility of replacement cells in response to externally applied cues, despite the significance of their spatial positioning within host tissue.

Our previous studies have shown that external cues, such as electric fields and chemical gradients, can induce migratory behaviors of SCs in microfluidic channels [39,49]. The microfluidic eye facsimile system, or μ-Eye, developed in this project applied electrical and chemotactic fields to guide the migration of replacement SCs into spherical, hydrogel facsimiles (or phantoms) of a whole eye. For proof of concept, eye facsimiles were synthesized from alginate, a hydrogel used in ophthalmology to provide controlled chemical release within biomimetic environments (Figure 3) [74]. Similarly, SCs were modeled using cultured, retinal precursors that expressed markers for the protein coding genes PAX6 and OTX2 (Figure 2), which are widely used in selecting transplantable cells [33,75].

The μ-Eye contains an inner electrode assembly that applies electric fields across eye facsimiles and an outer set of fluidic reservoirs that provide chemotactic stimulus by generating stable concentration gradients. Reservoirs were designed to maintain large, fluidic volumes for multi-day testing, and the electrode assembly can apply a range of electrical fields to stimulate SC motility (Figure 5). This design facilitates the application of electric fields currently used in animal studies and in the clinic, such as treatments with biochemical agents [76,77] and low level electric fields [78,79]. Electrical activity is known to play an important role in the formation and connectivity of neural circuits, where EF can modify synaptic connectivity, the structure of neuronal projections, and induce changes in gene expression, protein synthesis, and intrinsic excitability (reviewed in [80,81]). The ability to apply EF stimulation within the µ-Eye system therefore integrates a significant component that has not been fully explored therapeutically in the retina. Electrical and chemical transport across facsimiles were modelled numerically (Figure 6 and Figure 7) and validated experimentally to ensure a controlled and defined environment for testing (Figure 3). Furthermore, μ-Eye fabrication utilized conventional PDMS micro-molding and 3D printing (reviewed in [82,83]) to enable affordable, benchtop production (Figure 4) that aids medical collaboration and translational study.

The μ-Eye represents the first bioengineering system to integrate microfluidic environments with models of whole, enucleated eyes [72]. This is a significant step towards using in vitro and ex vivo technologies to aid development of in vivo strategies for transplantation, such as the use of EF and/electrodes [84], biomaterials [85], or pharmacological injection [77]. While a variety of in vitro and ex vivo platforms have been developed to examine SC behaviors (reviewed in [82,86]), the μ-Eye can bridge microfluidics with ocular explants to build hybrid, quantitative platforms for transplantation study in adult tissue environments. Furthermore, although dimensions of this project accommodated adult, murine eye facsimiles, fabrication of the μ-Eye is readily scaled to study facsimiles of different animal models. Future work will additionally adapt the system to examine SC migration within whole, enucleated eyes obtained from healthy animal specimens and disease models [87].

The μ-eye applied external chemotactic gradients and electric fields across facsimiles, individually and in parallel. The values of external fields were selected using previous work, from both our group and others, that have demonstrated SC migration towards the stimuli [22,39,49,88,89]. The results illustrated that both the average numbers of motile cells, N_T_, and their respective penetration depths within facsimiles, PD, were smaller for SDF stimulation than for the EF or EC fields (Table 1). The different stimuli were also seen to affect the distribution or final positioning of SCs within eye facsimiles. Subsequent data analyses of PD values used migration quartiles, Q, (shown in the box plots of Figure 8) to identify discrete sub-groups of SCs with different motilities and changes therein in response to external stimulus (Table 2). First, all stimuli produced nearly identical percentages of low motility cells (Q_1_) despite differences in penetration depth and numbers of motile cells. This suggests that some sub-population of SCs may always respond to external stimulus with low motility. Such data is consistent with published motility models that indicate that differences in internal signaling pathways and/or protein expression can occur across cells of the same lineage and/or type to influence migration [90,91]. Second, measurements of PD increased dramatically in response to combinatory EC fields, exceeding those measured using either SDF or EF stimulus, individually. Moreover, the percentage of SCs exhibiting elevated motility (Q_4_) also increased significantly during EC stimulation but remained largely unchanged between SDF and EF stimulation. This heightened, EC-induced migration suggests cross-talk between signaling pathways that regulate chemotactic and electrotactic motility (Reviewed in [92,93]) and point to exciting and unexplored applications of external fields for SC motility. Moreover, mechanisms of EC-induced motility can be elucidated using quantitative bioengineering platforms and hold promise to advance migration-based strategies for cell replacement therapy.

## 5. Conclusions

The μ-Eye system facilitates the study of the migratory behaviors of SCs within facsimiles, or phantoms, of adult eyes. The μ-Eye can be adapted to apply multiple external fields, use facsimiles and/or enucleated eyes from different animal models, and examine behaviors of SCs modified with specific proteins and/or transcription factors to promote migration. The system, thereby, provides a platform to explore new strategies to improve the outcomes of transplantation in the adult retina and in the nervous system more broadly.

## Figures and Tables

**Table 1:** Summary of the parameters used to describe cell migration within eye facsimiles per stimulation with chemotactic fields (SDF), electric fields (EF), and electro-chemotactic (EC) fields. The average, total number of motile cells measured within facsimiles is denoted by N_T_, the average penetration depth of motile cells is represented by PD_AVG_, the total range of PD values is denoted by R_T_, and the normality of the cell distribution is represented by the D’Agostino-Pearson *p*-value, *p*_NORM_.

	SDF	EF	EC
N_T_ (cells)	35 ± 2	55 ± 4	101 ± 4
PD_AVG_ (μm)	10.1 ± 5.9	125 ± 68.5	247 ± 123
Range (μm)	(2–20)	(25–225)	(40–440)
R_T_ (μm)	18	200	400
*p*_NORM_	0.12	0.88	<0.001

**Table 2:** Sub-groups of cell motility within eye facsimiles per different stimulus conditions of chemotactic fields (SDF), electric fields (EF), and combined electro-chemotactic (EC) fields. The average, total number of cells measured within facsimiles is denoted by N_T_. Cells with low motility were defined as those with penetration depths in the lowest quartile (Q_1_) of the respective motility range. Cells with average motility migrated penetration depths within the second and third quartiles (Q_2_, Q_3_) of the motility range, while cells with elevated motility penetrated eye facsimiles with the largest depths in the highest quartile (Q_4_). Data represents the average percentage of each motility sub-group across all tests per stimulus condition.

	SDF	EF	EC
Number of Motile Cells, N_T_	35 ± 2	55 ± 4	101 ± 4
Low Motility (Q_1_)	20.0%	21.8%	21.8%
Average Motility (Q_2_, Q_3_)	71.4%	67.3%	49.5%
Elevated Motility (Q_4_)	8.6%	10.9%	28.7%
